# Mucinous carcinoma originating from uterine adenomyosis: a case report

**DOI:** 10.1186/s13256-023-03772-w

**Published:** 2023-02-06

**Authors:** Satoshi Ohira, Ryota Tachibana, Sayaka Yasaki, Koji Tsunemi, Natsuki Uchiyama, Eri Ikeda, Kenji Sano

**Affiliations:** 1 Department of Obstetrics and Gynecology, Iida Municipal Hospital, 438 Yawatamachi, Iida, 395-8502 Japan; 2 Department of Pathology, Iida Municipal Hospital, 438 Yawatamachi, Iida, 395-8502 Japan

**Keywords:** Mucinous carcinoma, Adenomyosis, Case report

## Abstract

**Background:**

Uterine adenomyosis is rarely a precursor of malignant tumors, but the most frequent histological subtype is endometrioid carcinoma. We observed a rare case of mucinous carcinoma originating from uterine adenomyosis.

**Case presentation:**

A 63-year-old Japanese woman presented to our hospital with lower abdominal pain. She had no atypical genital bleeding. Ultrasound demonstrated thickening of the entire uterine wall, but the endometrium was not thick. Magnetic resonance imaging demonstrated an enlarged uterus with thickening of the entire uterine wall, suggesting adenomyosis. On the basis of the specimen of endocervical curettage, adenocarcinoma originating from the endometrium was suspected. Total abdominal hysterectomy and bilateral salpingo-oophorectomy were performed to confirm the diagnosis. Macroscopically, the resected enlarged uterus had no nodules and exudation of mucin was observed from the cut surface of the thickened myometrium. The surface of the endometrium was smooth. On histological examination, mucinous carcinoma invaded almost the entire myometrium. Adenomyotic lesions were distributed focally in the uterine wall, and transition from adenomyotic glandular epithelium to mucinous carcinoma was detected within several foci. Although adenocarcinoma cells proliferated adjacent to the endometrium, the primary endometrial epithelium was atrophic without atypia. Throughout the myometrium, the mucinous carcinoma cells proliferated and floated in dilated lymph vessels with abundant mucin pools. We diagnosed this case as mucinous carcinoma originating from adenomyosis. Although the patient received 11 courses of intravenous adjuvant chemotherapy, she died of disease 18 months after the first operation.

**Conclusion:**

As only one case of mucinous carcinoma originating from adenomyosis has been reported to date, this is the second case report of mucinous carcinoma. Moreover, an abnormal manner of proliferation with marked lymphatic permeation of the tumor cells throughout the myometrium was observed.

## Background

The ovary is the most common site of endometriosis, and endometriosis-associated ovarian cancer accounts for 0.3–1.0% of endometriosis [1]. The histological types of endometriosis-associated ovarian cancers are predominantly endometrioid and clear cell carcinoma, whereas serous and mucinous carcinoma are rare [2]. Uterine adenomyosis, a type of extraovarian endometriosis, is rarely a precursor of malignant tumor, but the most frequent histological subtype is endometrioid carcinoma [3]. We report a rare case of mucinous carcinoma originating from uterine adenomyosis. The current case exhibited an abnormal manner of proliferation with marked lymphatic permeation of the tumor cells throughout the myometrium, and this abnormal manner has not been reported to our knowledge.

## Case presentation

**Fig. 1:**
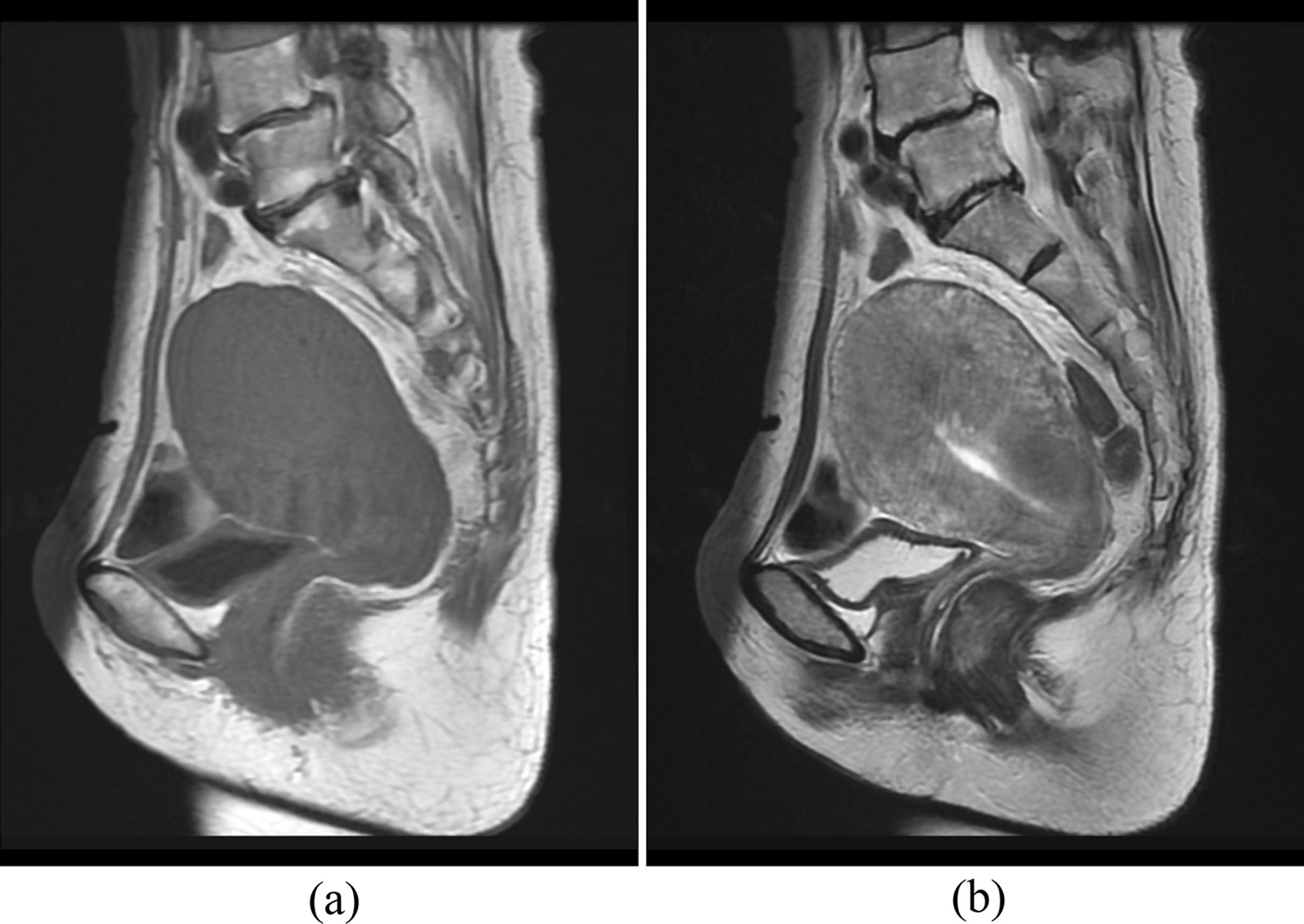
Magnetic resonance imaging demonstrated an enlarged uterus with thickening of the entire uterine wall, suggesting adenomyosis. Both T1-weighted (**a**) and T2-weighted (**b**) imaging revealed no uterine nodules

A 63-year-old postmenopausal Japanese woman (gravida 2, para 2) presented to the internal medicine of our hospital with a 2-month history of lower abdominal pain. Although uterine adenomyosis was noted in her forties, it was not followed up. She had no atypical genital bleeding. As the serum carbohydrate antigen 19-9 (CA19-9) level was high at 64,868 U/mL, close inspection of the pancreas, gallbladder, liver, and gastrointestinal tract was performed. Although gallbladder adenomyomatosis was detected, no malignant diseases were observed in these organs. Abdominal computed tomography (CT) revealed an enlarged uterus; therefore, she was referred to the department of gynecology. Ultrasound demonstrated thickening of the entire uterine wall, but the endometrium was not thick. Cytological testing of the endocervix revealed atypical glandular cells. Although it was not possible to pass the probe beyond the cervix, owing to the specimen of endocervical curettage, adenocarcinoma originating from the endometrium was suspected. Magnetic resonance imaging (MRI) demonstrated an enlarged uterus with thickening of the entire uterine wall, suggesting adenomyosis. Both T1- and T2-weighted imaging revealed no uterine nodules (Fig. 1). Three weeks later from the measurement with internal medicine, the serum CA19-9 level was markedly high at 115,950 U/mL. The serum cancer antigen 125 (CA125) level was high at 113.7 U/mL, whereas the carcinoembryonic antigen level was normal. No metastatic lesions were found on chest and abdominal CT.

**Fig. 2:**
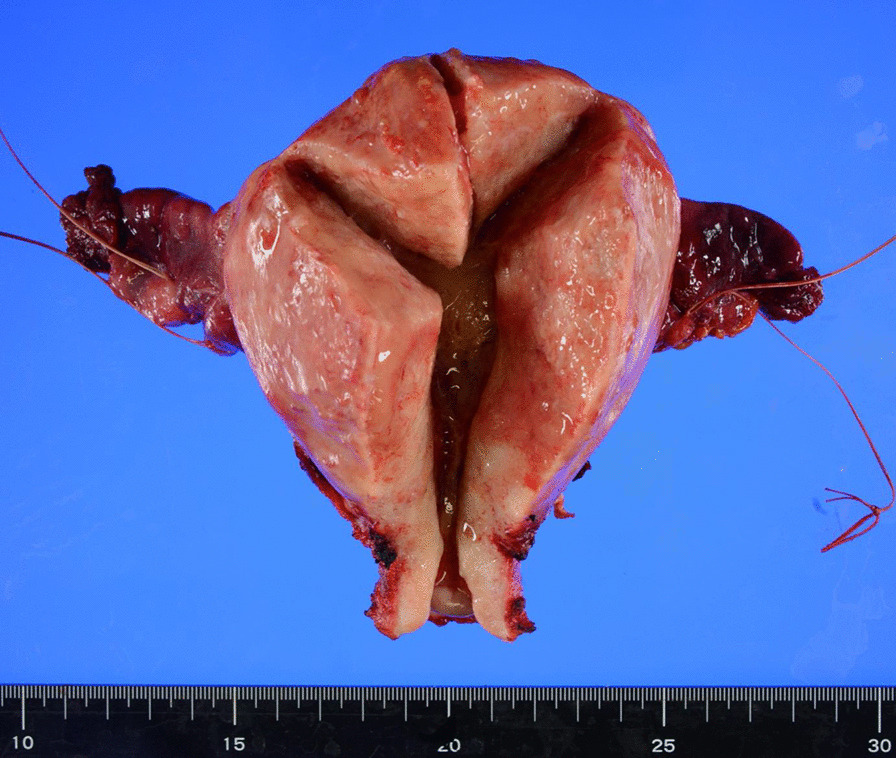
Macroscopically, the resected enlarged uterus had no nodules and the surface of the endometrium was smooth

Total abdominal hysterectomy and bilateral salpingo-oophorectomy were performed to confirm the diagnosis. Intraoperative ascitic cytology was negative, and there was no peritoneal dissemination. Macroscopically, the resected enlarged uterus had no nodules and exudation of mucin was observed from the cut surface of the thickened myometrium. The surface of the endometrium was smooth, and the bilateral ovaries were unremarkable (Fig. 2).

**Fig. 3:**
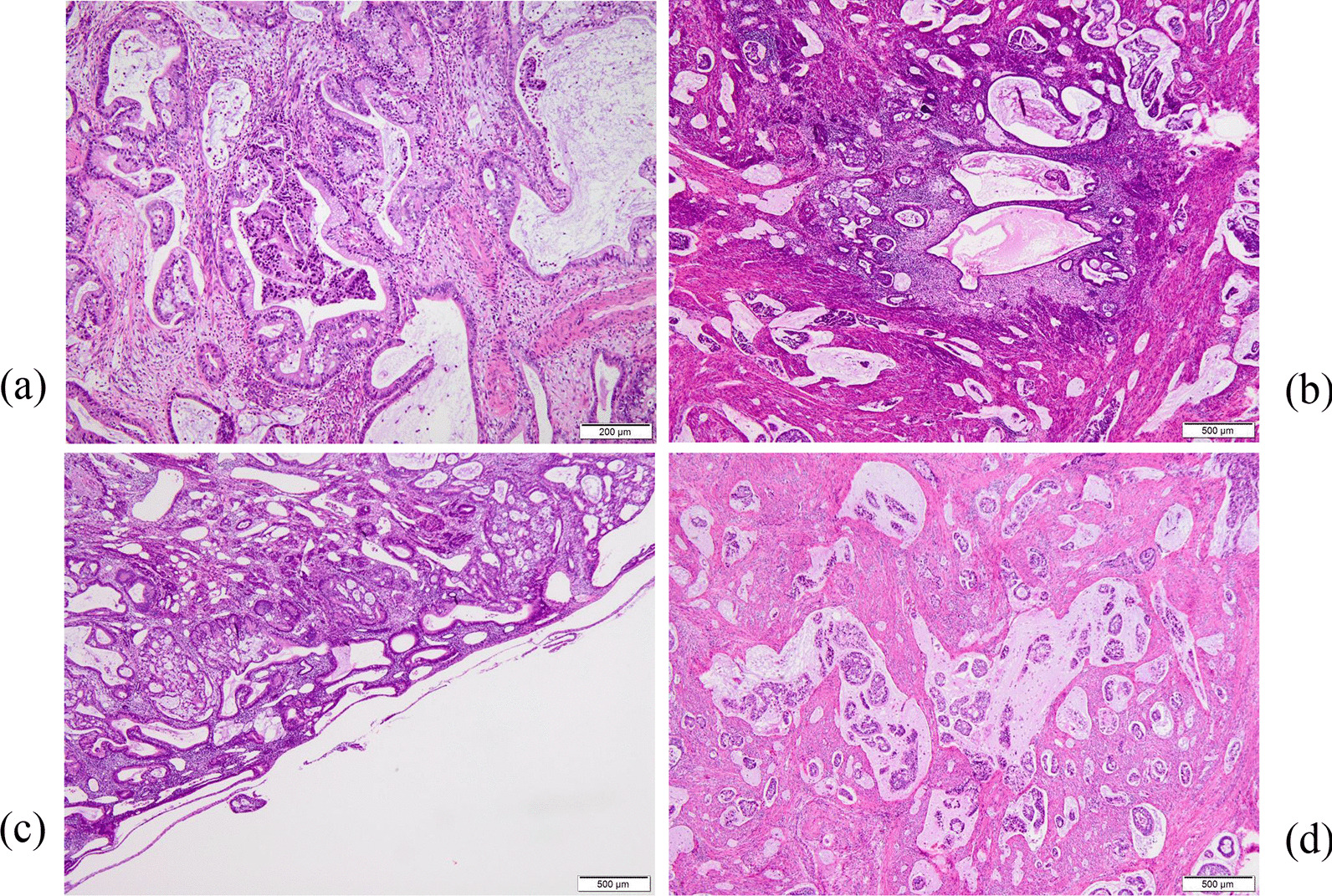
Histological findings of the resected uterus (hematoxylin and eosin staining). **a** Mucinous carcinoma invaded the myometrium and the tumor cells had a cribriform pattern composed of mucin-producing columnar epithelium. **b** Adenomyotic lesions were distributed focally in the uterine wall, and the transition from adenomyotic glandular epithelium to mucinous carcinoma was detected within several foci. **c** Although adenocarcinoma cells proliferated adjacent to the endometrium, the primary endometrial epithelium was atrophic without atypia. **d** The mucinous carcinoma cells proliferated and floated in dilated lymph vessels with abundant mucin pools

On histological examination, mucinous carcinoma (not otherwise specified; NOS) invaded almost the entire myometrium (Fig. 3a). The tumor cells had a cribriform pattern composed of mucin-producing columnar epithelium. The nuclei of the tumor cells were hyperchromatic; mitotic figures were sporadically observed. Adenomyotic lesions were distributed focally in the uterine wall, and transition from adenomyotic glandular epithelium to mucinous carcinoma was detected within several foci (Fig. 3b). Although adenocarcinoma cells proliferated adjacent to the endometrium, the primary endometrial epithelium was atrophic without atypia (Fig. 3c). Throughout the myometrium, the mucinous carcinoma cells proliferated and floated in dilated lymph vessels with abundant mucin pools (Fig. 3d). Microscopic metastatic lesions were observed in both ovaries. Endocervical epithelium and stroma of the uterine cervix were free of malignancy. Immunohistochemically, the adenocarcinoma cells were positive for p53 and CA19-9, but negative for estrogen receptor and p16.

According to the International Federation of Gynecology and Obstetrics (FIGO 2008) staging, we diagnosed this case as stage IIIA mucinous carcinoma originating from adenomyosis, pT3aN0MX. The patient received intravenous adjuvant chemotherapy (paclitaxel 175 mg/m^2^ and carboplatin AUC6). After six courses of chemotherapy, the serum CA19-9 and CA125 levels decreased to 36 U/mL and 7.4 U/mL, respectively.

Three months after the last course of chemotherapy, the serum CA19-9 level increased to 455 U/mL, and pelvic CT revealed swelling of left common iliac lymph node. Although the progressive disease was noted, the patient underwent five more courses of intravenous chemotherapy (paclitaxel 175 mg/m^2^ and carboplatin AUC6) in consideration of the patient’s desire. However, the degree of swelling of the lymph node on CT did not decrease and the left pelvic lymph nodes were resected. Microscopically, metastasis of adenocarcinoma was observed in the left common iliac lymph nodes. After a month of the second operation, CT revealed large swelling of paraaortic and mediastinal lymph nodes. The patient died of disease 18 months after the first operation.

## Discussion

Malignant neoplasms originating from uterine adenomyosis are rare. Koshiyama *et al.* reported that adenocarcinoma originating from adenomyosis accounted for 0.7% of all uterine body carcinomas treated by hysterectomy [4]. The most frequent histological subtype of malignant lesions is endometrioid carcinoma [3]. In our literature review, we found 23 well-documented cases of other histological subtypes as follows: 4 clear cell carcinomas [3–6], 4 serous carcinomas [4, 7, 8], 6 serous endometrial intraepithelial carcinomas [8, 9], 6 adenosarcomas [10–15], and 1 case each of adenosquamous carcinoma [16], carcinosarcoma [17], and mucinous carcinoma (minimal deviation adenocarcinoma; MDA) [18]. Therefore, the current case is the second case of mucinous carcinoma originating from uterine adenomyosis. Moreover, the current case (mucinous carcinoma, NOS) is distinguished from the reported case of mucinous carcinoma, gastric type (MDA).

The diagnostic criteria for carcinoma developing from adenomyosis were proposed by Colman and Rosenthal as follows [19]: (i) the carcinoma should be absent from the endometrium and elsewhere in the pelvis; (ii) the carcinoma should be observed arising from the epithelium of the areas of adenomyosis and not invading it from another source; and (iii) endometrial stromal cells should be surrounding the aberrant glands to support the diagnosis of adenomyosis. Our case does not strictly meet Colman’s criteria because the specimen of preoperative endocervical curettage suggested adenocarcinoma originating from the endometrium. However, we consider the diagnosis consistent with that of mucinous carcinoma originating from adenomyosis because of the observable transitions between adenomyosis and mucinous carcinoma.

In the current case, the tumor cells exhibited an abnormal manner of proliferation, that is, the mucinous carcinoma cells diffusely proliferated and floated in dilated lymph vessels with abundant mucin pools throughout the myometrium. Demarcated nodules were not observed on preoperative MRI or included in macroscopic findings of the resected uterus. Many cases of malignant tumors originating from adenomyosis exhibited demarcated lesions in the myometrium [6, 7, 15, 17, 20], and abnormal proliferation, as observed in our case, has not been reported to our knowledge. Moreover, the preoperative serum CA19-9 level in our case was markedly high at 115,950 U/mL. One reason for the high serum CA19-9 level was the marked mucin production from carcinoma cells. Although our patient underwent intravenous chemotherapy for the recurrent tumor in the pelvis, the treatment option might be radiotherapy in a case of recurrent mucinous carcinoma [21].

The prognosis of endometrial carcinoma originating from adenomyosis remains controversial. Machida *et al.* described that endometrial carcinoma arising in adenomyosis (EC-AIA) group (*n* = 46) had a significantly decreased 5-year disease-free survival (DFS) rates compared with endometrial cancer coexisting with adenomyosis (EC-A) group (*n* = 350) (72.2% versus 85.5%, *p* = 0.001) [22]. Meanwhile, Chao *et al.* studied a population with endometrial endometrioid carcinoma and described that the 5-year DFS rates were 96% in the group of EC without adenomyosis (*n* = 1043), 91% in the EC-A group (*n* = 230), and 100% in the EC-AIA group (*n* = 28) (*p* = 0.045) [23].

## Conclusions

We described a rare case of mucinous carcinoma originating from uterine adenomyosis. This case demonstrated an abnormal manner of proliferation with marked lymphatic permeation of the tumor cells throughout the myometrium. Further accumulation of cases is needed to clarify the pathogenesis and behavior of mucinous carcinoma originating from adenomyosis.

## Data Availability

All data presented in this report are included in this article.

## Abbreviations

CA19-9: Carbohydrate antigen 19-9
CT: Computed tomography
MRI: Magnetic resonance imaging
CA125: Cancer antigen 125
NOS: Not otherwise specified
MDA: Minimal deviation adenocarcinoma
EC-AIA: Endometrial carcinoma arising in adenomyosis
EC-A: Endometrial cancer coexisting with adenomyosis
DFS: Disease-free survival

## References

[1] Munksgaard PS, Blaakaer J (2012). The association between endometriosis and ovarian cancer: a review of histological, genetic and molecular alterations. Gynecol Oncol.

[2] Yoshikawa H, Jimbo H, Okada S, Matsumoto K, Onda T, Yasugi T (2000). Prevalence of endometriosis in ovarian cancer. Gynecol Obstet Invest.

[3] Baba A, Yamazoe S, Dogru M, Ogawa M, Takamatsu K, Miyauchi J (2016). Clear cell adenocarcinoma arising from adenomyotic cyst: a case report and literature review. J Obstet Gynaecol Res.

[4] Koshiyama M, Suzuki A, Ozawa M, Fujita K, Sakakibara A, Kawamura M (2002). Adenocarcinomas arising from uterine adenomyosis: a report of four cases. Int J Gynecol Pathol.

[5] Ohta Y, Hamatani S, Susuki T, Ikeda K, Kiyokawa K, Shiokawa A (2008). Clear cell adenocarcinoma arising from a giant cystic adenomyosis: a case report with immunohistochemical analysis of laminin-5 gamma2 chain and p53 overexpression. Pathol Res Pract.

[6] Hirabayashi K, Yasuda M, Kajiwara H, Nakamura N, Sato S, Nishijima Y (2009). Clear cell adenocarcinoma arising from adenomyosis. Int J Gynecol Pathol.

[7] Izadi-Mood N, Samadi N, Sarmadi S, Eftekhar Z (2007). Papillary serous carcinoma arising from adenomyosis presenting as intramural leiomyoma. Arch Iran Med.

[8] Lu B, Chen Q, Zhang X, Cheng L (2016). Serous carcinoma arising from uterine adenomyosis/adenomyotic cyst of the cervical stump: a report of 3 cases. Diagn Pathol.

[9] Abushahin N, Zhang T, Chiang S, Zhang X, Hatch K, Zheng W (2011). Serous endometrial intraepithelial carcinoma arising in adenomyosis: a report of 5 cases. Int J Gynecol Pathol.

[10] Oda Y, Nakanishi I, Tateiwa T (1984). Intramural Müllerian adenosarcoma of the uterus with adenomyosis. Arch Pathol Lab Med.

[11] Gollard R, Kosty M, Bordin G, Wax A, Lacey C (1995). Two unusual presentations of Müllerian adenosarcoma: case reports, literature review, and treatment considerations. Gynecol Oncol.

[12] Jha P, Ansari C, Coakley FV, Wang ZJ, Yeh BM, Rabban J (2009). Case report: imaging of Müllerian adenosarcoma arising in adenomyosis. Clin Radiol.

[13] Early HM, McGahan JP, Naderi S, Lamba R, Fananapazir G (2015). Müllerian adenosarcoma: a malignant progression of adenomyosis? Pictorial review with multimodality imaging. J Ultrasound Med.

[14] Lee SY, Park JY (2017). A rare case of intramural adenosarcoma arising from adenomyosis of the uterus. J Pathol Transl Med.

[15] Talia KL, Naaman Y, McCluggage WG (2021). Uterine adenosarcoma originating in adenomyosis: report of an extremely rare phenomenon and review of published literature. Int J Gynecol Pathol.

[16] Kay S, Frable WJ, Goplerud DR (1988). Endometrial carcinoma arising in a large polypoid adenomyoma of the uterus. Int J Gynecol Pathol.

[17] Kiuchi K, Hasegawa K, Kanamori A, Machida H, Kojima M, Fukasawa I (2016). Carcinosarcoma arising from uterine adenomyosis: a case report. J Obstet Gynaecol Res.

[18] Abiko K, Baba T, Ogawa M, Mikami Y, Koyama T, Mandai M (2010). Minimal deviation mucinous adenocarcinoma (‘adenoma malignum’) of the uterine corpus. Pathol Int.

[19] Colman HI, Rosenthal AH (1959). Carcinoma developing in areas of adenomyosis. Obstet Gynecol.

[20] Motohara K, Tashiro H, Ohtake H, Saito F, Ohba T, Katabuchi H (2008). Endometrioid adenocarcinoma arising in adenomyosis: elucidation by periodic magnetic resonance imaging evaluations. Int J Clin Oncol.

[21] Duzguner S, Turkmen O, Kimyon G, Duzguner IN, Karalok A, Basaran D (2020). Mucinous endometrial cancer: clinical study of the eleven cases. North Clin Istanb.

[22] Machida H, Maeda M, Cahoon SS, Scannell CA, Garcia-Sayre J, Roman LD (2017). Endometrial cancer arising in adenomyosis versus endometrial cancer coexisting with adenomyosis: are these two different entities?. Arch Gynecol Obstet.

[23] Chao X, Wu M, Ma S, Tan X, Zhong S, Bi Y (2020). The clinicopathological characteristics and survival outcomes of endometrial carcinoma coexisting with or arising in adenomyosis: a pilot study. Sci Rep.

